# Changes in the choroidal thickness in reproductive-aged women with iron-deficiency anemia

**DOI:** 10.1186/s12886-015-0163-3

**Published:** 2015-12-29

**Authors:** Erhan Yumusak, Aydin Ciftci, Selim Yalcin, Cemile Dayangan Sayan, Nevin Hande Dikel, Kemal Ornek

**Affiliations:** Department of Ophthalmology, Kırıkkale University School of Medicine, Kırıkkale, Turkey; Department of Internal Medicine, Kırıkkale University School of Medicine, Kırıkkale, Turkey; Department of Medical Oncology, Kırıkkale University School of Medicine, Kırıkkale, Turkey; Department of Obstetrics and Gynecology, Kırıkkale University School of Medicine, Kırıkkale, Turkey; Kirikkale University Medical Faculty Hospital District Tahsin Duru Akdeniz Caddesi No: 14, Yahsihan/Kırıkkale, 71450 Turkey

**Keywords:** Choroidal thickness, Iron-deficiency anemia, Enhanced-depth imaging optical coherence tomography

## Abstract

**Background:**

The aim of this study was to investigate the potential significance of the central macular thickness (foveal thickness-FT) and choroidal thickness (CT) in the eyes of patients with iron-deficiency anemia, the most common form of the anemia, via enhanced-depth imaging optical coherence tomography (EDI-OCT). We also investigated whether such changes might serve as an early indicator of underlying hematological disease.

**Methods:**

This prospective clinical study compared 96 female patients with iron-deficiency anemia and 60 healthy female control subjects. The macular and choroidal thicknesses in the temporal and nasal subfoveal areas were measured using enhanced-depth imaging optical coherence tomography (EDI-OCT) at 500 and 1500 microns and in five different regions (FCT, T_1500_, T_500_, N_500_, and N_1500_).

**Results:**

The mean ages of the patients and healthy controls were 34.08 ± 10.39 years and 32.29 ± 8.28 years, respectively (*P* =0.232). There were no significant changes in macular thickness between the groups (225.58 ± 19.76 vs. 222.45 ± 13.51, *P* =0.2). The choroidal thickness was significantly reduced in the patient group relative to the controls at all measured points (foveal choroidal thickness, *P* = 0.042; nasal–500 microns, *P* = 0.033; temporal–500 microns, *P* = 0.033; and temporal–1500 microns, *P* = 0.019). At some points, the choroidal thickness findings correlated with the hemoglobin values (temporal–500 microns, *r* = −0.287, *P* = 0.001; nasal–500 microns, *r* = −0.287, *P* = 0.005; nasal–1500 microns, *r* = −0.245, *P* = 0.016; and temporal–1500 microns, *r* = −0.280, *P* = 0.06).

**Conclusions:**

Patients with iron-deficiency anemia had a significantly reduced choroidal thickness.

## Background

The development of optical coherence tomography (OCT) technology has revolutionized the diagnostic, monitoring, and therapeutic approaches to many retinal and systemic diseases. Spectral domain OCT offers improved axial resolution (3 μm), reduces examination times, and accordingly reduces both patient exposure and artifacts by allowing 19,000 A-scans per second. The latest development in OCT technology, swept-source longer wavelength OCT, better penetrates the retinal pigment epithelium (RPE) and thus allows better visualization of the choroid; however, its use is currently limited to research purposes.

Similar to swept-source OCT, enhanced-depth imaging (EDI), which was recently introduced by Spaide et al. [1], provides greater penetration through the RPE and allows accurate in vivo deep choroidal imaging and measurement [2]. Changes in choroidal thickness (CT) have been found to correlate with several factors and systemic conditions such as smoking, changes in arterial pressure, hemodialysis, obstructive sleep apnea syndrome, and systemic sclerosis [3–7].

According to the World Health Organization (WHO), the overall global prevalence of iron-deficiency anemia is 25 %, with rates of 12.7 %, 30.2 %, and 41.8 % among adult men, women of reproductive age, and pregnant women, respectively [8]. Infants have the highest prevalence of iron-deficiency anemia, followed by pregnant women and women of reproductive age. In women of reproductive age, the risk of iron-deficiency anemia may accompany a range of important causes of morbidity and mortality [9, 10]. These conditions are the most important cause of menorrhagia in patients with iron-deficiency anemia [11, 12].

In this study, we investigated the potential significance of macular thickness and CT in the eyes of patients with iron-deficiency anemia, the most common form of anemia. We also considered the possibility that such changes might serve as an early indicator of underlying hematological disease. Using semi-automated choroidal segmentation software, we measured changes in CT in the eyes of women of reproductive age with iron-deficiency anemia in comparison with a control group.

## Methods

This prospective clinical study was conducted between 2014 and 2015 in accordance with the tenets of the Declaration of Helsinki. The trial protocol was approved by the Local Ethical Committee of the Kırıkkale University. Trial registration was requested on October 12, 2015 (decision no:01/04). All patients and control subjects voluntarily participated in the study and signed an informed consent form prepared according to the ethical protocol.

The patient group consisted of patients with a diagnosis of iron-deficiency anemia who were followed by the Hematology Clinic, whereas the control group consisted of healthy adult women of reproductive age. This study included only adult women of reproductive age to avoid the effects of gender, age, and hormonal status. The exclusion criteria were a previously diagnosed systemic or chronic disease or condition (e.g., hypertension, smoking, ocular surgery) in one or both eyes, an axial length >24 ± 1.0 mm, and a refractive measurement exceeding 2.0 diopters.

Patients with a previous diagnosis of iron-deficiency anemia were selected randomly for inclusion in the patient group. The study included 96 eyes from 96 patients followed in this manner, and only the patients right eyes were evaluated to avoid intra-individual bias. In the control group, 60 eyes from 60 patients were also evaluated and fully assessed. Each patient was subjected individually to visual acuity, slit-lamp biomicroscopy, intraocular pressure, and fundus examinations.

**Fig. 1:**
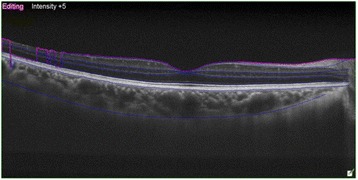
Choroid boundaries were manually drawn by a technician with software assistance

**Fig. 2:**
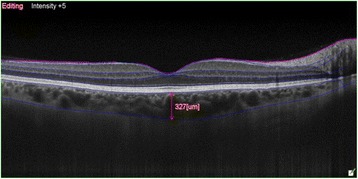
The choroidal thickness was measured manually with software assistance

CT and macular thickness measurement was performed on an EDI-OCT scanning system (OCT Advance Nidek RS-3000; Nidek Co. Ltd., Gamagori, Japan). In all patients, the central macular thickness (FT) was measured in the right eye prior to evaluation. Patients subsequently underwent EDI-OCT scanning. Technicians then manually drew the choroid and sclera boundaries with assistance from software programs (Fig. 1). These boundaries limited the Bruch membrane between the subfoveal points (FCT) to 500 or 1500 microns in the nasal (N_500_, N_1500_) and 500 or 1500 microns in the temporal regions (T_500_, T_1500_) for CT measurements (Fig. 2). Choroid and sclera demarcation was performed independently by two experienced technicians. The averages of the obtained values were used.

SSPS statistical software (SPSS for Windows, version 22.0; SPSS, Inc., Chicago, IL, USA) was used for the statistical analysis. The results of the descriptive analysis are presented as numbers, percentages, means, medians, and standard deviations. The paired *t* test and chi-square test were used to compare variables between the patient and control groups, and Pearson’s correlation coefficient was used to evaluate correlations. A multiple linear regression analysis (forward) was used to determine confounding factors among the variables. A *P* value < 0.05 was considered statistically significant.

## Results

The anemia group consisted of 96 female patients of reproductive age. The mean age was 34.08 ± 10.39 years (range: 18–49 years). The control group included 60 patients with a mean age of 32.2 9 ± 8.28 years (range: 17–49 years). The groups did not differ significantly with respect to age (*P* =0.232). Patients with anemia had a mean hemoglobin level of 9.59 ± 1.18 (range: 6.90–11). The mean duration of anemia was 4.18 ± 3.51 years (range: 1–15 years).

Forty-nine patients with a mean age of 33.86 ± 10.21 years were in the follicular phase of the menstrual cycle. Forty-six patients with a mean age of 34.32 ± 10.68 years were in the luteal phase. There were no significant differences between the patients in each phase with respect to age, hemoglobin level (10.09 ± 1.32 vs. 10.687 ± 1.55), and CT (*P* = 0.8, *P* = 0.7, and P > 0.05, respectively; (Table 1).

**Table 1:** Comparison of patients in two different phases of menstrual cycle (*t*-test) (LP = Luteal Phase, FP = Follicular phase; std.dev: standart deviation; FCT: choroidal thickness at fovea; N_500_, choroidal thickness at 500 μm nasal to the fovea; N_1500_, choroidal thickness at 1500 μm nasal to the fovea; T_500_, choroidal thickness at 500 μm temporal to the fovea; T_1500_, choroidal thickness at 1500 μm temporal to the fovea; FT: central macular thickness)

	N	Age	Hbvalue	FT	FCT	N_500_	N_1500_	T_500_	T_1500_
Mean FP	49	33.86	10.09	224.55	335.06	325.10	319.33	327.80	304.39
LP	47	34.32	10.34	226.66	324.47	318.51	315.04	310.47	288.06
Std. Dev. FP		10.21	1.33	19.23	63.42	81.71	81.71	88.59	92.29
LP		10.68	1.35	20.45	59.47	81.90	78.75	82.39	77.14
P		0.249	0.001 (with control groups)	0253	0.581	0.433	0.643	0.700	0.554

No statistically significant difference was observed between the groups with respect to FT (P > 0.05). When all measured CT points were compared, the anemia group had significantly lower values than the control group (Table 2).

**Table 2:** The numbers, mean, median ages, minimum, maximum values and choroidal thickness measurements of the study and control groups at baseline. FCT, choroidal thickness at fovea; N500, choroidal thickness at 500 μm nasal to the fovea; N1500, choroidal thickness at 1500 μm nasal to the fovea; T500, choroidal thickness at 500 μm temporal to the fovea; T1500, choroidal thickness at 1500 μm temporal to the fovea; FT: central macular thickness. (*t*-test)

	Age		Hb value	FT	FCT	N500	N1500	T500	T1500
Anemia	N	96	96	96	96	96	96	96	96
	Mean	34.08	10.21	225.58	329.88	321.88	317.23	319.31	296.40
	Median	34.50	10.65	220.00	326.50	315.00	319.00	321.00	294.00
	Std. Deviation	10.396	1.342	19.760	61.431	81,445	79.810	85.607	85.168
	Minimum	18	7	199	201	121	109	88	79
	Maximum	49	12	291	481	567	575	537	500
Control	N	60	60	60	60	60	60	60	60
	Mean	32.29	14.42	222.45	349.94	347.39	333.74	346.45	326.58
	Median	34.00	14.00	219.00	352.00	344.00	323.00	352.00	315.00
	Std. Deviation	8.283	0.95	13.513	59.064	56.718	55.549	63.100	66.157
	Minimum	17	13	200	217	247	231	231	205
	Maximum	49	16	256	442	508	458	525	474
	P	0.254	0.000	0.276	0.043	0.032	0.157	0.033	0.019

**Table 3:** Multiple linear regression analysis between choroidal thickness, age and hemoglobine values

		Beta(β)	P
N_500_	Hb	−0.290	0.003
	Age	−0.269	0.006
N_1500_	Hb	−0.249	0.009
	Age	−0.364	0.000
T_500_	Hb	−0.342	0.000
	Age	−0.289	0.002
T_1500_	Hb	−0.283	0.003
	age	−0.319	0.001
FCT	Hb	-	-
	Age	−0.291	0.004

*FCT* choroidal thickness at fovea; *N*_*500*_ choroidal thickness at 500 μm nasal to the fovea; *N*_*1500*_ choroidal thickness at 1500 μm nasal to the fovea; *T*_*500*_ choroidal thickness at 500 μm temporal to the fovea; *T*_*1500*_ choroidal thickness at 1500 μm temporal to the fovea; *Hb* Hemoglobin

In the patient group, no significant association was observed between the duration of anemia and the CT (P > 0.05). However, significant correlations were observed between some CT points and the hemoglobin values (temporal–500 micron, *r* = −0.287, *P* = 0.001; nasal–500 microns, *r* = −0.287, *P* = 0.005; nasal–1500 microns, *r* = −0.245, *P* = 0.016; temporal–1500 microns, *r* = −0.280, *P* = 0.06). Moreover, a statistically significant negative correlation was observed between age and CT (*P* <0.05).

We next assessed the independent effects of age and hemoglobin level on changes in CT through a multiple linear regression analysis. Only age independently affected the FCT (*p* = 0.004), whereas both age and the hemoglobin independently affected the N_500_, N_1500_, T_500_, and T_1500_ values. In other words, no overlap was observed between these factors; each CT point was affected independently by the hemoglobin level and age, with no potential confounding effect (*P* <0.05 for all; Table 3).

## Discussion

The choroid covers the outer retina and is among the most vascularized tissues in the body. This tissue supplies oxygen and nutrition to and provides temperature regulation for the retina. With respect to the retina, the choroid assumes the important roles of growth factor secretion and removal of retinal residues [13]. Both the structure and function of the choroidal tissue are essential factors ensuring a normal and functional retina. An abnormal choroidal blood volume, impaired photoreceptor function, and/or even a loss of blood flow can lead to injury [14]. The choroid features both sympathetic and parasympathetic innervation to regulate the choroidal blood flow, which is controlled by an autoregulatory mechanism [15].

The CT may be affected by age, axial length, and refractive errors [16, 17]. Diurnal changes in CT have also been reported [18]. The choroid is affected by changes in blood pressure and intraocular pressure through an autoregulatory mechanism [19]. Because the choroid features a rich vascular structure, these changes greatly affect the autoregulation of CT [20]. For example, Tanabe et al. demonstrated a significant relationship between the choroidal vessel diameter and CT [21]. Similarly, Vance et al. found that phoshodiesterase-5 inhibitors such as sildenafil citrate, which promotes smooth muscle relaxation, can lead to an increased CT [22].

In the current study, we found observed a significant reduction in CT in the patient group. In a review of the literature on this subject, we did not identify any previous research that would allow a direct comparison. Karaca et al. collected choroidal measurements at three points in their study of patients with obstructive sleep apnea syndrome and found no significant differences relative to control group measurements [6]. Pekel et al. evaluated choroidal changes in patients who had undergone cardiopulmonary bypass and also failed to detect any significant changes [4]. In addition, Ngo et al. presented a case report of high altitude-induced retinopathy in a patient with significantly increased CT [23].

Hematological diseases affect millions of patients worldwide and are a significant cause of both morbidity and mortality. Anemia consists of a group of hematologic disorders characterized by a decrease in the number of red blood cells and/or hemoglobin level. Factors such as hypoxia, venous stasis, vasospasm, and increased vascular permeability have been proposed in the pathogenesis of anemic retinopathy [24–26].

Therefore, we hypothesize that because the choroid is the most vascular area of the eye, the above factors also affect choroidal tissues in patients with iron-deficiency anemia.

In our study, we observed a significant correlation between hemoglobin levels and CT. Moreover, we observed an inverse correlation between age and CT. Ding et al. reported similar results with respect to CT in a study of areas outside the subfoveal wet area in subjects younger than 60 years of age but failed to detect a correlation between age and subfoveal CT [27].

In the Beijing Eye Study, 3468 individuals were examined longitudinally, and several parameters related to the subfoveal CT was assessed [28]. That study observed direct links between age or the degree of myopia and CT. However, the potential associations or lack thereof between blood pressure, ocular perfusion, smoking, and alcohol consumption with diabetes mellitus and arterial hypertension remain controversial. However, that study relied only on single-point measurements. Notably, choroidal volume measurements risk being subject to false information [29].

In our study, we did not observe any significant differences between the two menstrual cycle phases. Because the menstrual cycle has a short duration, it is possible that we were unable observe a hormonal effect, or that the hormonal status does not affect CT. We did observe significant but moderate or weak correlations between the hemoglobin levels and the CT values at some measured points. However, the previously observed inverse correlation between age and CT might have affected this correlation [27]. The patients ranged in age from 17 to 49 years. No significant difference was observed between the duration of anemia duration and CT. This suggests that changes in CT do not progress during the course of anemia.

## Conclusion

Increasing attention is being paid to information about the choroidal structure and function. We think that as future studies of patients with anemia reveal the possible effects of this condition on choroidal tissue, the observation of decreased CT at all measured points in patients with iron-deficiency anemia might suggest the inclusion of this value as a clinical measurement in this patient population.

## Abbreviations

EDI-OCT: enhanced-depth imaging optical coherence tomography
CT: choroidal thickness
FCT: choroidal thickness at fovea
N_500_: choroidal thickness at 500 μm nasal to the fovea
N_1500_: Choroidal thickness at 1500 μm nasal to the fovea
T_500_: choroidal thickness at 500 μm temporal to the fovea
T_1500_: choroidal thickness at 1500 μm temporal to the fovea
FT: foveal thickness (Central macular thickness)
LP: luteal Phase
FP: follicular phase
RPE: retinal pigment epithelium
WHO: World Health Organization
Hb: hemoglobin
